# Metabolomic Profiling of the Striatum in *Shank3* Knockout ASD Rats: Effects of Early Swimming Regulation

**DOI:** 10.3390/metabo15020134

**Published:** 2025-02-16

**Authors:** Yunchen Meng, Yiling Hu, Yaqi Xue, Zhiping Zhen

**Affiliations:** 1 Department of Physical Education and Research, China University of Mining and Technology—Beijing, Beijing 100083, China; zqt2410707005p@student.cumtb.edu.cn; 2 College of P.E and Sports, Beijing Normal University, Beijing 100875, China; xueyaqi555@mail.bnu.edu.cn

**Keywords:** *Shank3*, early swimming, striatum, metabolism, neurotransmitter

## Abstract

**Objectives:** This study aimed to investigate the regulatory impact of early swimming intervention on striatal metabolism in *Shank3* gene knockout ASD model rats. **Methods:** *Shank3* gene knockout exon 11–21 male 8-day-old SD rats were used as experimental subjects and randomly divided into the following three groups: a *Shank3* knockout control group (KC), a wild-type control group (WC) from the same litter, and a *Shank3* knockout swimming group (KS). The rats in the exercise group received early swimming intervention for 8 weeks starting at 8 days old. LC-MS metabolism was employed to detect the changes in metabolites in the striatum. **Results:** There were 17 differential metabolites (14 down-regulated) between the KC and WC groups, 19 differential metabolites (18 up-regulated) between the KS and KC groups, and 22 differential metabolites (18 up-regulated) between the KS and WC groups. **Conclusions:** The metabolism of striatum in *Shank3* knockout ASD model rats is disrupted, involving metabolites related to synaptic morphology, and the Glu and GABAergic synapses are abnormal. Early swimming intervention regulated the striatal metabolome group of the ASD model rats, with differential metabolites primarily related to nerve development, synaptic membrane structure, and synaptic signal transduction.

## 1. Introduction

Autism spectrum disorder (ASD) is a complex neurodevelopmental condition marked by social impairments, repetitive and stereotyped behaviors, and a spectrum of symptoms encompassing cognitive deficits, motor dysfunction, intellectual disability, and attention deficit hyperactivity disorder (ADHD) [1,2]. The global incidence of ASD has risen significantly, posing a major public health challenge that affects children’s well-being. According to a 2021 report by the Centers for Disease Control and Prevention (CDC), the prevalence of ASD among 8-year-olds was 1 in 44, representing a staggering 240% increase from 1 in 150 in 2000 [3]. In China, nearly 1% of children suffer from varying degrees of ASD, with the prevalence rate continuing to climb annually [4]. Given the high incidence and lifelong disability associated with ASD, early diagnosis and intervention are pivotal in its prevention and management [5].

The etiology of ASD is multifaceted, involving intricate interactions between genetic and environmental factors. While the precise causes remain unknown for at least 60% of ASD cases [6], extensive research has established that ASD originates in the early stages of brain development and persists throughout life. The genetic underpinnings of ASD are complex, characterized by widespread variations in genetic networks. Mutations or deletions in numerous genes, notably the *Shank3* gene, have been implicated in the pathogenesis of ASD [7,8]. The protein encoded by the *Shank3* gene serves as a vital scaffolding component in the postsynaptic density (PSD) of excitatory synapses, playing a crucial role in synapse formation and stabilization and the regulation of synaptic transmission. Disruptions in the *Shank3* gene can impair normal synaptic function, leading to aberrant neuronal connections and, subsequently, triggering ASD symptoms [9]. Consequently, the genetic knockout of *Shank3* has emerged as a valuable animal model for studying autism.

In recent years, research into the pathophysiology of ASD has intensified, leading to a growing interest in exercise intervention as a non-pharmacological treatment option. Swimming, a whole-body exercise that combines motor stimulation with multi-sensory stimulation from a rich environment, is recognized as an effective means of promoting neurodevelopment and neural plasticity [10]. Appropriate swimming exercise not only bolsters cardiopulmonary function and enhances physical fitness but also exerts positive effects on the central nervous system, improving neurotransmitter release and fostering synaptic plasticity [11]. Studies on swimming interventions for ASD patients have documented its beneficial impact on ASD symptoms. Fragala-Pinkham et al. reported that a 14-week swimming program improved cardiopulmonary endurance and physical fitness in children with disabilities, including those with ASD [12]. Pan’s research demonstrated that aquatic therapy significantly enhanced muscle strength, endurance, and social interaction skills in children with ASD [13]. Furthermore, Ennis et al. found that swimming interventions improved social, emotional, and learning outcomes in children with ASD [14]. These findings suggest that swimming exercise may ameliorate ASD symptoms by promoting neural plasticity and enhancing synaptic function. However, despite promising initial evidence, the specific mechanisms underlying the benefits of swimming intervention in ASD treatments remain incompletely understood. Particularly in the context of the *Shank3* gene knockout ASD animal model, further investigation is needed to determine whether swimming interventions can modulate striatal synaptic function to alleviate ASD symptoms. The striatum, a key component of the central nervous system, plays a crucial role in regulating various functions, including movement, cognition, and emotion. Structural and functional abnormalities in the striatum have been widely documented in individuals with ASD [15]. Therefore, exploring the effects of swimming interventions on striatal synaptic function in *Shank3* gene knockout rats with ASD is of paramount importance in elucidating the mechanisms underlying the therapeutic benefits of swimming.

In a previous study, *Shank3^−^*^/*−*^ rats exhibited social impairments and stereotypic behaviors. Notably, these behavioral abnormalities showed improvement when the rats underwent early swimming exercise interventions [9]. A transcriptomic analysis of the striatum revealed differential gene expression related to synaptic structure and function between the *Shank3^−^*^/*−*^ and *Shank3^+^*^/*+*^ rats, further highlighting the differences between the *Shank3^−^*^/*−*^ control rats and those subjected to swimming interventions [10]. The altered striatal transcriptome is likely to exert widespread influences on neurotransmitter release within the striatum. Abnormalities in neurotransmission, including glutamate, GABA, dopamine, serotonin, opioids, and oxytocin, have been reported in ASD patients [16,17].

Metabolites are small molecules produced as byproducts of enzymatic reactions and may serve as final or intermediate products. The abundance and composition of metabolites within a tissue directly reflect changes in the genome, transcriptome, and proteome [18]. Consequently, an emerging area of central nervous system (CNS) research has focused on the study and characterization of the metabolome. Among the metabolites relevant to CNS research are energy substrates, neurotransmitters, neurochemicals, and structural lipids. In this study, we employed a metabolomic approach to further investigate the effects of *Shank3* knockout and early swimming interventions on the metabolite profiles in rat striatum.

## 2. Materials and Methods

### 2.1. Experimental Animals and Grouping

Male specific-pathogen-free (SPF) Sprague Dawley (SD) *Shank3^−^*^/*−*^ rats at the age of 8 days old were obtained from the Peking University School of Medicine. The germline deletion of *Shank3*, specifically targeting exon 11–21, was generated using CRISPR-Cas9 technology [19]. The *Shank3^−^*^/*+*^ rats, both male and female, were generated by backcrossing *Shank3^−^*^/*−*^ rats with *Shank3^+^*^/*+*^ SD rats. Subsequently, the *Shank3^−^*^/*+*^ rats were bred to obtain both SD *Shank3^+^*^/*+*^ and *Shank3^−^*^/*−*^ rats for the experimental groups. The rats were randomly allocated into the following three groups based on their genotypes and treatments: the *Shank3^−^*^/*−*^ control group (KC; n = 3), *Shank3^+^*^/*+*^ littermates (WC; n = 3), and the *Shank3^−^*^/*−*^ swimming group (KS; n = 3).

The rats were housed in cages containing 3 animals each, with free access to food and water, in a room maintained at a temperature of 20–25 °C, with 45–55% humidity and a 12-h light/dark cycle. All animal procedures and care were conducted in strict accordance with the ethical standards and protocols outlined by the National Institutes of Health (US) [cite PMID 21595115] and were approved by the Biomedical Ethics Committee of Peking University (ethics number LA2021552). After the experiment, all the animals were euthanized by intraperitoneal injection of barbiturates.

### 2.2. Early Swimming Exercise Program

**Figure 1:**
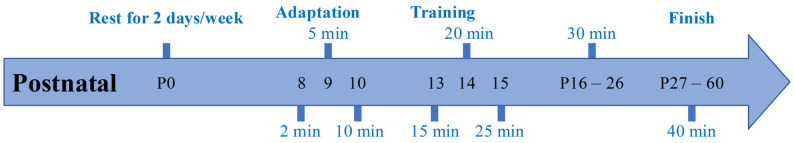
Early swimming intervention program [10]. The protocol included swimming from postnatal day (P) 8 to 60 and always at the same time (17:00 to 18:00), with rest periods for two consecutive rest days by week. The pups practiced swimming for 2, 5, and 10 min on P8, 9, and 10, respectively, and rested on P11 and 12. On P13, the rats swam for 15 and 20 min on P14 and 25 min on P15. From P16 to 26, the rats swam for 30 min/day, 5 times per week. From days 27 to 60, the rats swam for 40 min/day, 5 times per week.

The swimming protocol employed in this study was adapted from a prior study by Muniz [20] and was performed on the rats in the KS group as depicted in Figure 1. An initial adaptation to swimming was implemented for the rats in the KS group, as illustrated in Figure 1 [10,21]. The initial acclimation to swimming took place during the post-natal days (PND) 8–10 in a cage measuring 485 × 350 × 200 mm that was filled with water maintained at a temperature of 32 ± 1 °C. The duration of each session was 2 min on PND 8, 5 min on PND 9, and 10 min on PND 10. On PND8, the water level was at the height of the rats’ legs. On PND9, the water level was at the height of the rats’ stomachs. On PND10, the water level was at the height of the rats’ necks. Starting from PND 13, the swimming intervention was conducted in a circular tank (150 cm in diameter and 100 cm in height) filled with temperature-controlled water (32 ± 1 °C) to a depth of 50 cm, a setup that remained consistent for the remainder of the experiment. The duration of each session was 15 min on PND 13, 20 min on PND 14, and 25 min on PND 15. Between PND16–26, the rats engaged in 30 min of swimming per day, with 5 consecutive days of swimming followed by 2 days of rest per week. The duration of the swimming sessions was extended to 40 min per day from PND 27 to 60.

### 2.3. Striatal Metabolite Analysis

#### 2.3.1. Chemicals

This experiment utilized a variety of solutions and reagents. Liquid chromatography-mass-spectrometry-grade methanol and acetonitrile were both purchased from Thermo (Waltham, MA, USA). We sourced 2-chlorophenylalanine from Aladdin (Carlsbad, CA, USA). High-performance liquid-chromatography-grade formic acid was obtained from TCI (Tokyo, Japan). Liquid-chromatography-grade ammonium formate was purchased from Sigma (Darmstadt, Germany). The experimental water was ultrapure water with a resistivity of 18.2 MΩ/cm, and it was purified using a Millipore ultrapure water system (Burlington, NJ, USA). Additionally, ACS-grade chloroform was sourced from Wokai (Beijing, China). These solutions and reagents provided a solid guarantee for the accurate conduct of the experiment.

#### 2.3.2. Metabolite Extraction

The rats were euthanized using pentobarbital sodium at a dose of 150 mg/kg, followed by the careful removal of their brains. To access the striatum, forceps were applied to the medial cortex. Approximately 100 mg (±2%) of striatal tissue was then transferred into a high-throughput tissue grinder (Meibi, Jiaxing, China) containing 1 mL of tissue extraction buffer (composed of 75% methanol/chloroform 9:1 and 25% H_2_O) kept at −20 °C. Three steel balls with diameters of 3 mm were added to facilitate the grinding process. The tissue was ground at 50 Hz for 60 s, which was repeated twice, followed by sonication at room temperature for 30 min. Subsequently, the samples were centrifuged at 12,000 rpm for 10 min at 4 °C, and 850 μL of the resultant supernatant was carefully extracted and concentrated under vacuum until it reached a dry state. For further analysis by liquid chromatography-mass spectrometry (LC-MS), 200 μL of a solution consisting of 50% acetonitrile in 2-chloro phenylalanine (20 ppm) was added to 200 μL of 50% acetonitrile. The resulting mixture was then filtered through a 0.22 μm membrane.

#### 2.3.3. Chromatographic Conditions

The liquid chromatography (LC)-tandem mass spectrometry (MS/MS) system consists of a Waters UPLC (Dublin, Ireland) separation module coupled to a Thermo LTQ (Waltham, MA, USA) ion trap mass spectrometer. An ACQUITY UPLC^®^ HSS T3 (2.1 × 150 mm, 1.8 µm, Waters, Dublin, Ireland) column was used with a sample temperature of 8 °C at room temperature and 40 °C at a flow rate of 0.25 mL/min. A gradient elution was performed using a mobile phase consisting of 0.1% formic acid in water (A2) and 0.1% formic acid in acetonitrile (B2). The gradient elution procedure consisted of the following steps: ① 0–1 min, 2–50% B2; ② 9.5–14 min, 50–98% B2; ③ 14–15 min, 98% B2; ④ 15–15.5 min, 98–2% B2; and ⑤ 15.5–17 min, 2% B2.

#### 2.3.4. Mass Spectrometric Conditions

An electrospray ionization source (ESI) was employed using both the positive and negative ionization modes. The positive ion spray voltage was set at 4.80 kV, while the negative ion spray voltage was set at 4.50 kV. The sheath gas was maintained at 45 arb, and an auxiliary gas was used at 15 arb. The capillary temperature was set at 325 °C. A full scan was conducted at a resolution of 60,000, with a scan range of 89–1000 for positive ions and 114–1000 for negative ions. High-energy collision-induced dissociation (HCD) was applied for secondary cleavage, utilizing a collision voltage of 30 eV. Additionally, dynamic exclusion was implemented to eliminate unnecessary MS/MS information.

#### 2.3.5. Data Analysis

Peak identification was performed using the XCMS package (version v3.8.2) of the Proteowizard software (version v3.0.8789) and R (version v3.3.2). Additionally, dynamic exclusion was implemented to eliminate unnecessary MS/MS information. This analysis provided essential information, including the mass-to-nucleus ratio (*m*/*z*), retention time (rt), and peak area (intensity), which was then normalized using sum peak area normalization. The complete dataset can be accessed at MetaboLights (MTBLS8577).

Metabolite identification was conducted utilizing public spectral databases such as HMDB, MassBank, LipidMaps, mzCloud, and KEGG, as well as the proprietary standard compound library established by Nuomi Metabolomics. The parameter was set with a mass deviation of less than 30 ppm to obtain qualitative metabolite results. The specific principle involved determining the molecular weights of the metabolites based on the *m*/*z* of the parent ions in the primary mass spectrum. Molecular formulas were predicted using information on the mass deviation and adduct ions, and then they were matched against the databases to achieve primary metabolite identification. Simultaneously, for the metabolites with detected secondary mass spectra listed in the quantification results, fragment ions and other relevant information were matched with each metabolite in the databases to achieve secondary metabolite identification.

The supervised pattern recognition method was used, and a partial least squares-discriminant analysis (PLS-DA) was performed on each group of data. The parameters of R2X, R2Y, and Q2 were extracted. For data processing, the pheatmap package in R was used for scaling and plotting, applying agglomerative hierarchical clustering with the Euclidean distance as the distance metric. Hierarchical clustering was performed based on the relative values of the metabolites under different experimental conditions, and the results were presented in heat maps. A log10 transformation was applied to the raw data, and clustering for both rows and columns was carried out using the Canberra algorithm. The metabolites were considered significantly different at a *p*-value < 0.05 and a variable importance in projection score (VIP) of >1. The MetPA database was used to analyze the relevant metabolic pathways for each group of differential metabolites, the hypergeometric test algorithm was used for the data analysis, and relative-betweenness centrality was used for the pathway topology analysis.

## 3. Results

### 3.1. LC-MS Analysis of the Striatal Metabolome

In this study, LC-MS metabolomics was employed to analyze the metabolite compositions and contents in striatal tissue samples obtained from three distinct groups of rats. These groups included *Shank3^−^*^/*−*^ rats that underwent early-life swimming exercise intervention, as well as *Shank3^−^*^/*−*^ and *Shank3^+^*^/*+*^ control rats that did not receive the intervention.

**Figure 2:**
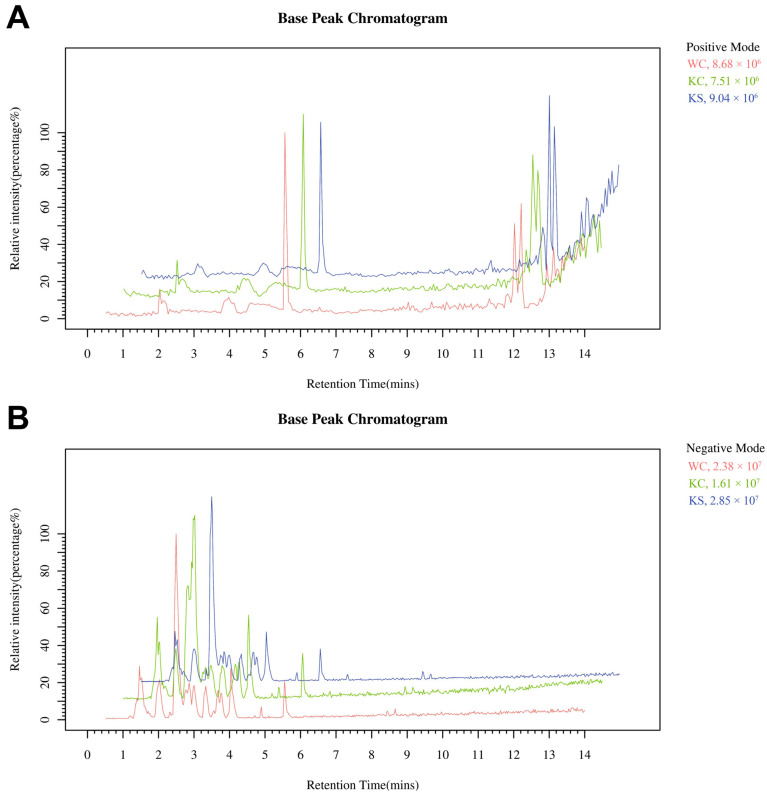
Ion-mode base peak chromatogram. (**A**) A chromatogram of the positive ion mode base peak, and (**B**) a chromatogram of the negative ion mode base peak.

The BPC trend was consistent for all QC samples, indicating perfect reproducibility and reliable data (Figure 2). Remarkably, the retention times and intensity profiles of the sample peaks exhibited excellent reproducibility, indicating both the high quality of the samples and the reliability of our analytical methodology. In addition, a visual inspection of the chromatograms revealed discernible differences among the various experimental groups, warranting further in-depth analysis and investigation.

### 3.2. PLS-DA

**Figure 3:**
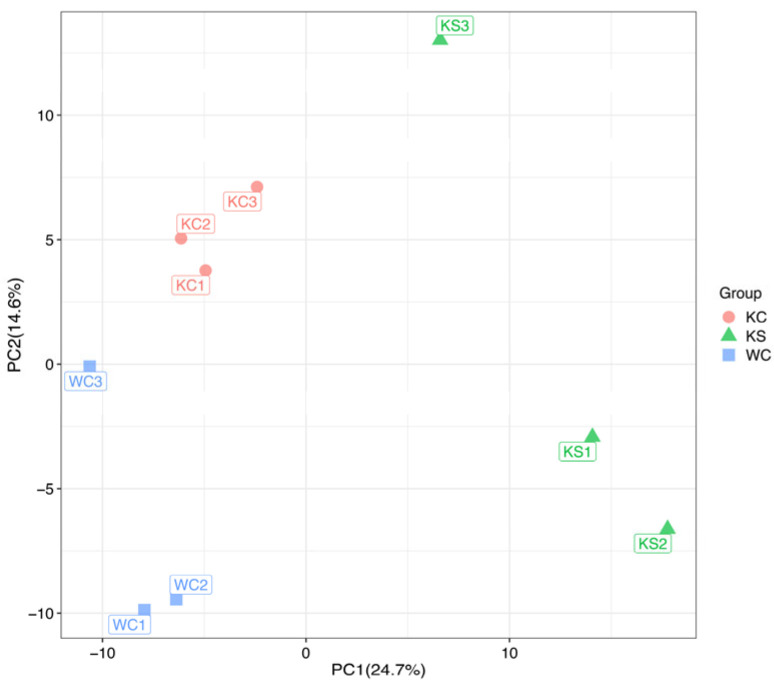
Plots of the PLS-DA scores, loadings, and replacement tests for each comparison group in the positive and negative ion modes.

The effectiveness of the current PLS-DA model was assessed using permutation graphs to determine the risk of overfitting. The results, as illustrated in Figure 3, indicated significant differences in the combined positive and negative ions as well as the metabolite profiles among the WC, KC, and KS groups. Specifically, the combined positive and negative ion PLS-DA model exhibited R^2^Y (cum) = 1 and Q^2^ (cum) = 0.66. These values indicated a highly robust and reliable fit, with R^2^Y (cum) = 1 suggesting a perfect explanation of the variance in the response variable by the model and Q^2^ (cum) = 0.66 indicating a good predictive power and model validity.

### 3.3. Differential Metabolite Screening

**Figure 4:**
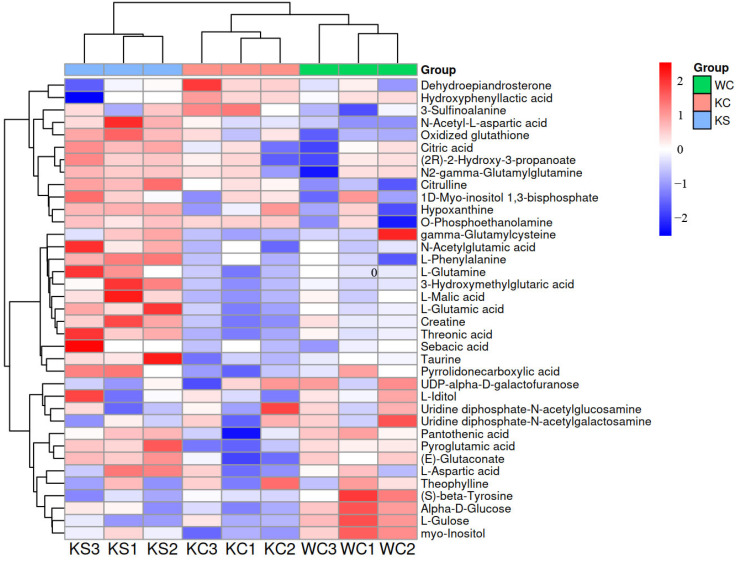
Heat map of the ionic pattern differences in the metabolites.

Differential metabolite screening was conducted using stringent criteria, specifically, a *p*-value < 0.05 and a VIP of >1. These criteria were applied to both the positive and negative ion pattern metabolites in the following three rat striatal metabolism groups: WC, KC, and KS. Subsequently, heat maps representing the differential metabolites of the ions were generated (Figure 4). Following an annotation analysis of the ion pattern differential compounds, a comprehensive list of the differential metabolites was compiled. In the KC and WC groups, a total of 17 differential metabolites were identified, with 14 metabolites showing down-regulation. In the KS and KC groups, 19 differential metabolites were detected, with 18 metabolites exhibiting up-regulation. Similarly, in the KS and WC groups, 22 differential metabolites were found, with 18 metabolites displaying up-regulation. A summary of these findings is presented in Table 1.

### 3.4. Metabolic Network Analysis of the Differential Metabolites

**Figure 5:**
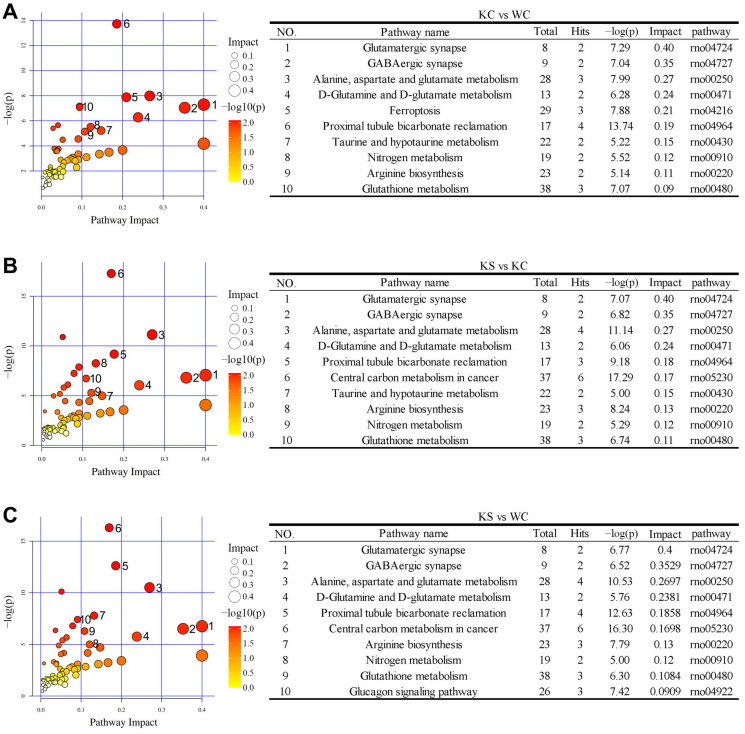
Differential metabolite enrichment pathways in the striatum samples of each comparison group. (**A**) A bubble diagram of the differential metabolite enrichment pathway in the KC vs. WC groups; (**B**) a bubble diagram of the differential metabolite enrichment pathway in the KS vs. KC groups; and (**C**) a bubble diagram of the differential metabolite enrichment pathway in the KS vs. WC groups.

The metabolic pathway analysis of the differential metabolite enrichment is depicted in Figure 5. In the KC and WC groups, the enrichment of the differential metabolites was observed in several key pathways, including the glutamatergic synapse, the GABAergic synapse, alanine, aspartate and glutamate metabolism, D-glutamine and D-glutamate metabolism, ferroptosis, proximal tubule bicarbonate reclamation, taurine and hypo taurine metabolism, nitrogen metabolism, arginine biosynthesis, and glutathione metabolism. Comparatively, the KS vs. KC and KS vs. WC groups exhibited similar patterns of metabolite enrichment pathways, as observed in the KC vs. WC group. However, the KS vs. KC group displayed an additional increase in the central carbon metabolism in cancer pathway, while the KS vs. WC group exhibited an increase in the glucagon-signaling pathway.

## 4. Discussion

### 4.1. Metabolomic Analysis of the Striatum in Shank3 Knockout Rats

ASD represents a complex set of neurodevelopmental disorders with etiological origins rooted in a combination of genetic and environmental factors. Metabolic abnormalities have been identified in several monogenic animal models of ASD, and changes in metabolite levels are emerging as crucial indicators of altered pathway activity in the pathogenesis of ASD. For instance, Rett syndrome, a monogenetic form of ASD resulting from inactivation of the X-linked MECP2 gene encoding the transcription factor methyl cytosine phosphoribosyl guanine binding protein, has been studied extensively [22]. A metabolic analysis of brain extracts from Mecp2-deficient mice, conducted using 1H-NMR, has revealed significant alterations in the D-glutamine and glutamate metabolism [23]. Fragile X syndrome (FXS), the most common genetic cause of ASD, is associated with intellectual disability and results from a recessive X-linked mutation in the fragile X messenger ribonucleoprotein 1 (FMR1) gene [24]. Comprehensive metabolomic studies on the Fmr1-KO mouse model of FXS have demonstrated region-specific metabolic profiles, with the cerebellum and cerebral cortex being particularly affected [25]. These studies have highlighted disruptions in various neurotransmitters, including GABA, glutamate, taurine, and acetylcholine. A metabolite set enrichment analysis (MSEA) revealed significant impacts on the metabolism of D-glutamine, D-glutamate, arginine, and proline [26]. Elevated levels of lipid oxides detected in the cortex of Fmr1-KO mice have indicated increased oxidative stress [26], consistent with previous observations [27,28]. Notably, this metabolic profile exhibited a striking overlap with the pattern detected in the Rett mouse model [23].

In this study, we conducted the first-ever metabolomic analysis of striatal brain regions in a *Shank3* knockout animal model. A comparison between the KC group and the WC group revealed that absence of *Shank3* led to alterations in 17 differential metabolites. Among these, 14 metabolites exhibited down-regulation while 3 metabolites showed up-regulation. Consistent with previous findings for the central differential metabolites observed in animal models of Rett and FXS syndromes, the striatum of the *Shank3* knockout rat model displayed disruptions in key metabolites, including glutamate, glutamine, and oxidized glutathione. These alterations suggest disturbances in synaptic signaling and an increase in oxidative stress. Furthermore, our analysis identified disruptions in metabolites such as taurine, threonine, tyrosine, and glucose, indicating potential abnormalities in neurodevelopmental processes. A functional enrichment analysis of these differential metabolites, combined with transcriptomics [10], revealed functional abnormalities associated with neurodevelopmental processes and oxidative stress. Notably, the disruptions in the GABAergic and glutamatergic synaptic function hint at an imbalance in excitatory-inhibitory (E-I) signaling, a factor closely linked to the pathogenesis of ASD [29].

Our analysis further revealed that several key metabolic pathways were enriched in the striatum of the *Shank3* knockout rats compared to the wild-type controls. These pathways, namely, the glutamatergic synapse and ferroptosis, hold significant biological relevance to the physiological conditions of ASD. The glutamatergic synapse pathway involves various metabolites crucial for glutamate synthesis, storage, release, and uptake [30]. Disruptions in glutamatergic signaling have been widely reported in ASD, leading to imbalances in E-I balance and synaptic transmission [31]. Our finding that the glutamatergic synapse pathway was enriched in the *Shank3* knockout rats aligned with previous studies showing alterations in glutamate-related metabolites in ASD models. SHANK3 proteins are essential for the formation and stabilization of synapses, particularly glutamatergic synapses [32]. Therefore, the absence of *Shank3* likely disrupts glutamate signaling, contributing to the ASD phenotype. The ferroptosis pathway, a form of cell death driven by iron-dependent lipid peroxidation, involves multiple metabolites that regulate cellular redox balance and lipid homeostasis [33]. Recent studies have implicated ferroptosis in the pathogenesis of several neurodevelopmental disorders, including ASD [34]. Oxidative stress, a hallmark of ASD, can trigger ferroptosis by overwhelming cellular antioxidant defenses [35]. Our results indicate that the ferroptosis pathway is enriched in *Shank3* knockout rats, suggesting that oxidative stress-induced cell death may play a role in the striatal abnormalities observed in this ASD model. The disruption of *Shank3*-mediated synaptic functions may lead to increased oxidative stress, promoting ferroptosis and contributing to neuronal damage and dysfunction.

### 4.2. Metabolomic Changes in the Striatum of Rats After Early Swimming Interventions

Previous research has explored the potential therapeutic benefits of swimming interventions in animal models of neurological disorders. These studies have unveiled possible mechanisms through which swimming can mitigate neurological disorders, including up-regulation of neurotrophic factors within the central nervous system [36,37,38], alterations in synaptic structural proteins [39], changes in synaptic receptor expression [37,40], modifications in neurotransmitter release [41], and adjustments in multiple pathways that affect synaptic plasticity [39,42,43].

In our earlier work, our team conducted transcriptomic studies demonstrating that early swimming interventions not only upregulated genes associated with neural development, synaptic transmission, and synaptic morphology but also modulated various signaling pathways [10]. To delve deeper into the changes in striatal neurotransmission in *Shank3* knockout rats following early swimming interventions and to understand the mechanisms behind the positive behavioral effects observed in animal models of ASD, we extended our previous findings by investigating alterations in striatal metabolomics after swimming interventions in *Shank3* knockout rats. After early swimming intervention, 19 metabolites were changed in the KS group compared with the KC group, of which 18 metabolites were up-regulated and only 1 metabolite was down-regulated. Crucial metabolites related to neurodevelopment, such as taurine and threonine, as well as signaling molecules like glutamate and glutamine, which were down-regulated in the striatum samples due to the *Shank3* knockout, were found to be restored following early swimming interventions. This indicated that early swimming interventions could mitigate aspects of abnormal neurodevelopment and synaptic signaling resulting from *Shank3* gene dysfunction. A subsequent pathway enrichment analysis of these differential metabolites revealed up-regulation in the metabolites associated with taurine and subtaurine metabolism, alanine and aspartate metabolism, arginine biosynthesis, D-glutamine and D-glutamate metabolism, and glutathione metabolism, all of which are linked to neurodevelopment and synaptic transmission, particularly in glutamatergic and GABAergic synapses. The modulatory effects of the early swimming interventions on neurodevelopment and E-I balance were further corroborated. Additionally, a combined analysis of the metabolomics and transcriptomics in the KS and KC groups unveiled functions related to energy metabolism, including glycolysis/gluconeogenesis, and functions related to ASD disease development, such as purine metabolism.

Furthermore, an analysis of the differential metabolites between the KS and WC groups was conducted, revealing 22 differential metabolites, with 18 exhibiting up-regulation. Of particular note, metabolites such as taurine, creatine, glutamine, and glutamate, which had been down-regulated in the striatum samples from the *Shank3* knockout rats, were found to be restored following early swimming interventions. These findings strongly suggest that abnormalities in related metabolites caused by *Shank3* knockout can be effectively reversed by early swimming interventions, potentially leading to improvements in neural development and signaling processes.

The differences in the metabolic enrichment pathways observed between the KS vs. KC and KS vs. WC groups provided deeper insights into the mechanisms by which early swimming interventions may alleviate ASD-like symptoms in *Shank3* knockout rats. In the KS vs. KC group comparison, the primary enrichment pathways included those related to synaptic function (e.g., glutamatergic synapse and GABAergic synapse), energy metabolism (e.g., central carbon metabolism in cancer), and antioxidant pathways (e.g., glutathione metabolism). The up-regulation of metabolites involved in the glutamatergic and GABAergic synapses suggests that early swimming interventions help restore the balance of excitatory and inhibitory neurotransmission, which is vital for normal brain function [44]. Moreover, the enrichment in the central carbon metabolism in the cancer pathway may have indicated enhanced energy metabolism in the striatum samples from the rats undergoing swimming exercise, potentially supporting neuronal recovery and synaptic plasticity [45]. The increase in the glutathione metabolism pathway could reflect a reduction in oxidative stress, a recognized factor in ASD pathogenesis [46]. In the KS vs. WC group comparison, a similar pattern of metabolic enrichment was observed, with additional emphasis on pathways such as glucagon signaling. The restoration of metabolites related to synaptic function and energy metabolism, as well as the reduction in oxidative stress, aligned with the improvements seen in the KS vs. KC comparison. The enrichment in the glucagon signaling pathway, which plays a role in glucose homeostasis and energy metabolism, may indicate that early swimming interventions not only improve synaptic function but also optimize overall metabolic health in the striatum [47]. These differences in the metabolic enrichment pathways have profound implications for the study’s outcomes. The restoration of synaptic function and energy metabolism pathways in the KS group, relative to both the KC and WC groups, suggests that early swimming interventions exert a therapeutic effect on the striatal metabolism of *Shank3* knockout rats. The up-regulation of antioxidants and the reduction in oxidative stress further support the neuroprotective role of swimming exercise in this ASD model.

Collectively, these findings indicate that early swimming interventions can effectively reverse some of the metabolic abnormalities caused by *Shank3* knockout, potentially leading to improvements in synaptic function, neuronal health, and, ultimately, behavioral outcomes in *Shank3* knockout rats. This not only offers a mechanistic explanation for the beneficial effects of swimming interventions in ASD but also identifies the key metabolic pathways that may serve as targets for future therapeutic interventions. Furthermore, the differences in metabolic enrichment between the KS vs. KC and KS vs. WC groups underscore the importance of utilizing both knockout and wild-type control groups in metabolomic studies. While comparisons with knockout controls are essential for isolating the effects of an intervention, comparisons with wild-type controls provide additional insights into the extent to which the intervention can restore normal metabolic function. Overall, the comparative analysis of the metabolic enrichment pathways in this study revealed that early swimming interventions have profound impacts on striatal metabolism in *Shank3* knockout rats, suggesting a potential therapeutic role for swimming exercise in ASD.

## 5. Conclusions

In summary, the metabolomic findings indicate that *Shank3* knockout results in abnormal striatal neurodevelopment and disrupted glutamatergic and GABAergic synaptic function in rats. Additionally, concerning neurotransmitter release, there is a potential disruption in the excitatory–inhibitory (E-I) balance of synaptic signaling. Early swimming interventions effectively reversed the down-regulation of some essential metabolites associated with neurodevelopment and synaptic signaling, which had been negatively impacted by *Shank3* knockout. This reversal could be a key factor contributing to the improvement in the behavior of *Shank3* knockout rats following early swimming interventions.

## Figures and Tables

**Table 1:** Differential metabolite profiles of the striatal tissue samples in the WC, KC, and KS groups.

No.	Metabolites	*m*/*z*	RT (s)	Formula	KEGG	Type	Fold Change		
							KC/WC	KS/KC	KS/WC
1	Taurine	124.01	89.03	C_2_H_7_NO_3_S	C00245	[M−H]−	0.92 *	1.23 *	1.13 *
2	Pyroglutamic acid	128.04	177.84	C_5_H_7_NO_3_	C01879	[M−H]−	0.88	1.57 *	1.39 *
3	(E)-Glutaconate	129.02	103.77	C_5_H_6_O_4_	C02214	[M−H]−	0.31 *	4.53 *	1.43 *
4	Creatine	130.06	99.11	C_4_H_9_N_3_O_2_	C00300	[M−H]−	0.88 *	1.29 *	1.14 *
5	L-Malic acid	133.01	119.43	C_4_H_6_O_5_	C00149	[M−H]−	0.84 *	1.62 *	1.36 *
6	Threonic acid	135.03	97.76	C_4_H_8_O_5_	C01620	[M−H]−	0.74 *	2.15 *	1.59 *
7	L-Glutamine	145.06	88.52	C_5_H_10_N_2_O_3_	C00064	[M−H]−	0.86 *	1.58 *	1.36 *
8	L-Glutamic acid	146.05	92.43	C_5_H_9_NO_4_	C00025	[M−H]−	0.86 *	1.53 *	1.32 *
9	3-Hydroxymethylglutaric acid	161.05	239.48	C_6_H_10_O_5_	C03761	[M−H]−	0.78 *	2.66 *	2.08 *
10	Myo-Inositol	161.05	556.35	C_6_H_12_O_6_	C00137	[M−H_2_O−H]−	0.61 *	1.25	0.76 *
11	L-Phenylalanine	166.09	280.18	C_9_H_11_NO_2_	C00079	[M+H]+	1.06	2.16 *	2.29 *
12	(2R)-2-Hydroxy-3-propanoate	166.98	105.63	C_3_H_7_O_7_P	C00197	[M−H_2_O−H]−	1.05	2.04 *	2.14 *
13	N-Acetyl-L-aspartic acid	174.04	151.21	C_6_H_9_NO_5_	C01042	[M−H]−	1.41 *	1.95 *	2.76 *
14	Alpha-D-Glucose	179.06	763.79	C_6_H_12_O_6_	C00267	[M−H]−	0.46 *	1.31	0.61 *
15	L-Iditol	181.07	665.47	C_6_H_14_O_6_	C01507	[M−H]−	0.76 *	1.21	0.92
16	(S)-beta-Tyrosine	181.07	808.76	C_9_H_11_NO_3_	C21308	[M]−	0.29 *	1.20	0.35 *
17	N-Acetylglutamic acid	188.06	200.43	C_7_H_11_NO_5_	C00624	[M−H]−	0.84	2.28 *	1.92 *
18	Citric acid	191.02	167.31	C_6_H_8_O_7_	C00158	[M−H]−	0.99	1.85 *	1.82 *
19	Sebacic acid	201.11	565.53	C_10_H_18_O_4_	C08277	[M−H]−	0.96	7.66 *	7.32 *
20	Pantothenic acid	218.10	294.11	C_9_H_17_NO_5_	C00864	[M−H]−	0.59 *	1.64 *	0.97
21	Gamma-Glutamylcysteine	248.96	81.79	C_8_H_14_N_2_O_5_S	C00669	[M−H]−	0.84 *	1.18	0.98
22	N2-gamma-Glutamylglutamine	274.10	101.94	C_10_H_17_N_3_O_6_	C05283	[M−H]−	1.10	1.77 *	1.95 *
23	Dehydroepiandrosterone	288.29	724.49	C_19_H_28_O_2_	C01227	[M]+	1.89 *	0.51 *	0.96
24	Oxidized glutathione	611.14	219.81	C_20_H_32_N_6_O_12_S_2_	C00127	[M−H]−	2.20 *	2.05 *	4.52 *
25	Theophylline	179.06	643.65	C_7_H_8_N_4_O_2_	C07130	[M−H]−	0.88	0.85	0.74 *
26	Hypoxanthine	135.03	170.48	C_5_H_4_N_4_O	C00262	[M−H]−	1.24	1.29	1.60 *

“*” indicates a significant difference between the two groups (*p* < 0.05).

## Data Availability

The complete dataset can be accessed at MetaboLights (MTBLS8577).
